# Architecture of the human NALCN channelosome

**DOI:** 10.1038/s41421-022-00392-4

**Published:** 2022-04-06

**Authors:** Lunni Zhou, Haobin Liu, Qingqing Zhao, Jianping Wu, Zhen Yan

**Affiliations:** 1 Fudan University, Shanghai, China; 2 Key Laboratory of Structural Biology of Zhejiang Province, School of Life Sciences, Westlake University, Hangzhou, Zhejiang China; 3 Westlake Laboratory of Life Sciences and Biomedicine, Hangzhou, Zhejiang China; 4 Institute of Biology, Westlake Institute for Advanced Study, Hangzhou, Zhejiang China

**Keywords:** Cryoelectron microscopy, Protein-protein interaction networks

## Abstract

NALCN regulates the resting membrane potential by mediating the Na^+^ leak current in neurons, and it functions as a channelosome in complex with FAM155A, UNC79, and UNC80. Dysfunction of the NALCN channelosome causes a broad range of neurological and developmental diseases called NALCN channelopathies in humans. How the auxiliary subunits, especially the two large components UNC79 and UNC80, assemble with NALCN and regulate its function remains unclear. Here we report an overall architecture of the human NALCN channelosome. UNC79 and UNC80 each adopt an S-shape super-helical structure consisting of HEAT and armadillo repeats, forming a super-coiled heterodimeric assembly in the cytoplasmic side, which may provide a scaffold for the binding of other potential modulators of the channelosome. The UNC79–UNC80 assembly specifically associates with the NALCN–FAM155A subcomplex through the intracellular II–III linker of NALCN. Disruptions of the interaction interfaces between UNC79 and UNC80, and between the II–III linker of NALCN and the UNC79–UNC80 assembly, significantly reduce the NALCN-mediated currents in HEK293T system, suggesting the importance of the UNC79–UNC80 assembly in regulating channelosome function. Cross-linking mass spectrometry analysis identified an additional calmodulin (CaM) bound in the carboxyl-terminal domain of NALCN. Our study thus provides a structural basis for understanding the unique assembly mechanism and functional regulation of the NALCN channelosome, and also provides an opportunity for the interpretation of many disease-related mutations in UNC80.

## Introduction

The sodium leak channel NALCN plays an important role in regulations of neuronal excitability, motor function, pain sensitivity, and circadian rhythm^1–4^. NALCN forms a unique complex termed NALCN channelosome with the auxiliary subunits FAM155A, UNC79, and UNC80^5–7^. The formation of the NALCN channelosome is critical to a fully functional Na^+^ conductance carried by NALCN^8^. Knockout of NALCN, UNC79, or UNC80 is lethal in mice^1,9,10^. In humans, variants of NALCN and the auxiliary subunits, especially UNC80, are linked to a variety of diseases known as NALCN channelopathies^11,12^, which could cause severe intellectual disabilities, such as infantile hypotonia with psychomotor retardation and characteristic facies (IHPRF)^11,13^ and congenital contractures of limbs and face, hypotonia and developmental delay (CLIFAHDD)^12,14,15^.

The ion-conducting subunit NALCN is topologically similar to Na_v_ and Ca_v_ channels, which contain four homologous repeats (I–IV) connected by intracellular linkers and belong to the 4 × 6 transmembrane helices (TM) channels within the superfamily of voltage-gated ion channels^16^. However, the auxiliary subunits of the NALCN channelosome are unique and share no sequence homology to the auxiliary subunits of other 4 × 6 TM channels. Among the auxiliary subunits, FAM155A may facilitate the folding and membrane translocation of NALCN through forming a stable subcomplex with NALCN^17–19^. UNC79 and UNC80 are large proteins that have been reported indispensable for neuronal localization in mice^9^ and robust circadian locomotor rhythms in *Drosophila*^6^. Previous studies indicate that UNC80 directly interacts with NALCN and forms a binding scaffold for UNC79 and other potential regulators such as the Src family of tyrosine kinases (SFKs)^20,21^. However, how UNC79 and UNC80 associate and regulate the function of NALCN remains obscure.

We have previously reported the structure of human NALCN–FAM155A subcomplex, revealing that FAM155A stabilizes NALCN by attaching to the extracellular loops of NALCN^17^. UNC79 and UNC80, however, were invisible in the previous structure, although they were co-expressed with NALCN and FAM155A. This may suggest that their association with NALCN–FAM155A subcomplex is not stable in vitro. In this study, we sought to elucidate the structure of the intact human NALCN channelosome, providing insights into its assembly and regulation mechanisms.

## Results

### Purification of the human NALCN channelosome

**Fig. 1:**
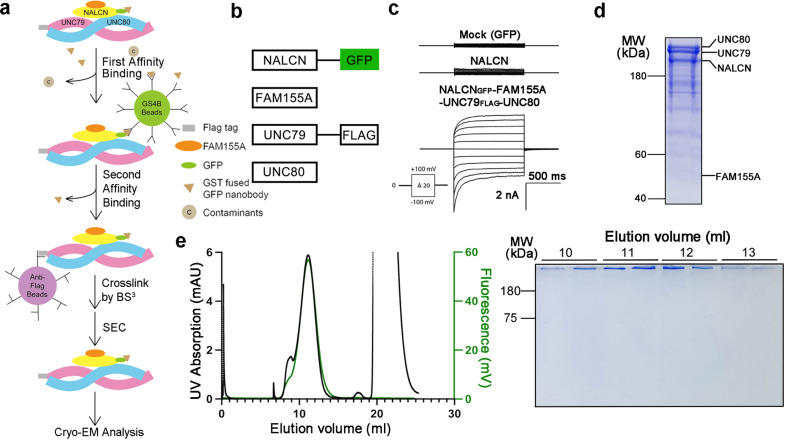
Purification and electrophysiological characterization of the human NALCN channelosome. **a** Schematic diagram of the purification of the human NALCN channelosome. **b** Constructs used for the NALCN channelosome expression and electrophysiological experiments. FAM155A and UNC80 are untagged. The carboxyl termini of NALCN and UNC79 were fused with a green fluorescent protein (GFP) and a 2× FLAG tag, respectively. **c** Representative current traces recorded from HEK293T cells expressing Mock (GFP), NALCN alone and the NALCN channelosome. **d** The protein sample visualized by Coomassie blue-stained SDS–PAGE gel after tandem affinity purification. The four channelosome components NALCN, FAM155A, UNC79, and UNC80 are labeled. **e** Size-exclusion chromatogram and corresponding SDS–PAGE result of the crosslinked NALCN channelosome sample. The monodisperse peak indicates good solution behavior of the protein sample.

To obtain the intact channelosome, we optimized the protein expression and purification procedures by fusing a GFP tag after NALCN and a 2× FLAG tag after UNC79, followed by a tandem-affinity purification step (Fig. 1a, b). The purified protein contains all four components, and the constructs used for purification record canonical NALCN currents in HEK293T cells (Fig. 1c, d; Supplementary Fig. S1). After the affinity columns, the eluted NALCN_GFP_–FAM155A–UNC79_FLAG_–UNC80 complex was further stabilized by BS^3^ cross-linking before the size-exclusion chromatography. The purified NALCN channelosome sample displays a monodisperse peak in gel filtration, which indicates the sample is homogeneous and suitable for structural elucidation by single particle cryo-electron microscopy (cryo-EM) study (Fig. 1e).

### Overall architecture of the NALCN channelosome

**Fig. 2:**
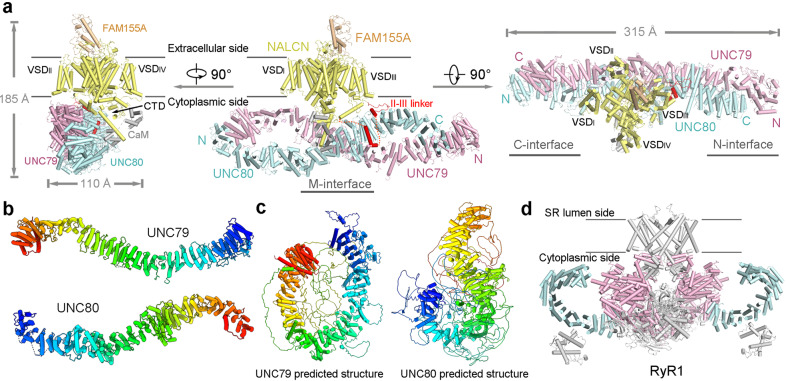
Overall architecture of the human NALCN channelosome. **a** Overall structure of the NALCN channelosome shown in three views. NALCN, FAM155A, UNC79, UNC80, and CaM are colored yellow, wheat, pink, cyan, and gray, respectively. The II–III linker of NALCN that interacts with the UNC79–UNC80 assembly is highlighted in red. The color scheme is applied in all figures. **b** UNC79 and UNC80 adopt an S-shape super-helical structure. The structures are rainbow colored with the N- and C-terminus in blue and red, respectively. **c** Overall structures of UNC79 and UNC80 predicted by AlphaFold2. The predicted structures form super-helical architecture consisting of HEAT and armadillo repeats. The overall shape is different from the experimental structure as shown in **b**. **d** Structure of RyR1 contains a huge super-helical scaffold in the cytoplasmic side (PDB: 3J8H). Only two diagonal protomers of the homo-tetrameric RyR1 are shown for visual clarity.

The channelosome particles display a characteristic triangular shape (Supplementary Fig. S2a–c). After 3D classification, the selected particles generated a final reconstruction at an overall resolution of 4.5 Å, which show discernable secondary structure features (Supplementary Figs. S2d–f, S3a). Local refinement further improves the best cytosolic region containing UNC79 N-half and UNC80 C-half to 3.3 Å, enabling side chain assignment of this region (Supplementary Fig. S3b, c). Despite moderate resolution of other areas, a reliable overall model of the channelosome can be built, facilitated by the NALCN–FAM155A subcomplex structure and the predicted structures of UNC79 and UNC80 by AlphaFold2 (Fig. 2a–c; Supplementary Tables S1, S2). The model was further validated by cross-linking mass spectrometry (XL-MS) analysis. Most of the cross-link pairs, including about 10 inter-subunit pairs, are highly consistent with the structure (Supplementary Fig. S4). In particular, a cross-link pair between NALCN–K1569 and CaM–K95 suggests that CaM is probably a physically interacting regulator of the channelosome that is co-purified from the HEK293F expression system (Supplementary Fig. S4d). Consistent with this analysis, an extra density near the carboxyl-terminal domain (CTD) of NALCN was observed in the cryo-EM map, which should belong to the co-purified CaM (Supplementary Fig. S3a).

Due to the presence of the massive cytosolic components UNC79 and UNC80, the channelosome has an overall dimension of 315 Å × 185 Å × 110 Å, much larger than the NALCN–FAM155A subcomplex (Fig. 2a). UNC79 and UNC80 form a heterodimeric supercoiled assembly that sits beneath NALCN near the voltage-sensing domain II (VSD_II_) side. The overall architecture of NALCN channelosome is reminiscent of an upside-down bamboo dragonfly, in which the UNC79–UNC80 assembly looks like the propeller wing and the NALCN–FAM155A subcomplex resembles the rotating shaft (Fig. 2a). This unique architectural feature implies a potential gating mechanism of NALCN, in which the gating of NALCN may be coupled with the conformational changes of the large cytosolic assembly. Consistent with this idea, 3D viability analysis of the cryo-EM dataset shows a large landscape of conformational dynamics of the UNC79–UNC80 assembly, whose motion is more like waving wings than spinning (Supplementary Video S1). The dynamic architectural characteristics of the UNC79–UNC80 assembly provide a molecular basis to achieve their regulations of NALCN gating.

### Structure of the UNC79–UNC80 assembly

**Fig. 3:**
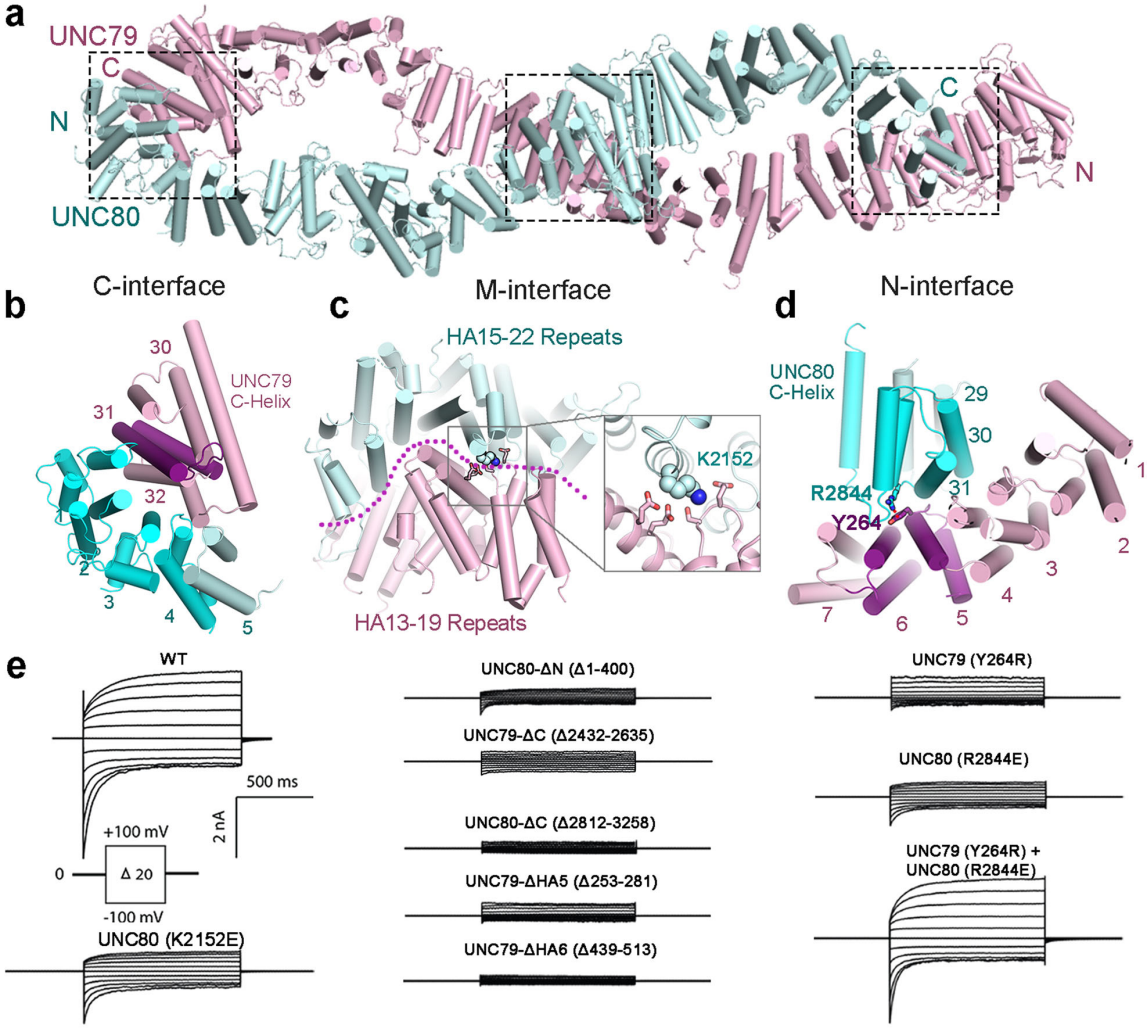
Architecture of the UNC79–UNC80 assembly. **a** Overall structure of the UNC79–UNC80 assembly shown in cartoon. Three interaction interfaces between UNC79 and UNC80 are labeled. **b**–**d** Close-up views of the interaction interfaces between UNC79 and UNC80: the C-interface (**b**), the M-interface (**c**), and the N-interface (**d)**. The numbers of the UNC79 and UNC80 HA repeats that form the corresponding interfaces are labeled. The side chains of the residues on the interfaces that are mutated and tested by electrophysiological experiments are labeled in color. **e** Representative current traces recorded from HEK293T cells expressing the WT NALCN channelosome and the constructs with indicated mutations or deletions on the interfaces between UNC79 and UNC80.

Both UNC79 and UNC80 adopt an overall S-shape super-helical assembly, which are different from their overall predicted structures by AphlaFold2, although the secondary structures are consistent (Fig. 2b, c). They are mainly formed by HEAT and armadillo (HA) repeats, 32 repeats of UNC79 and 31 repeats of UNC80, together forming a head-to-tail supercoiled assembly, which looks like a horizontal number ‘8’ (Figs. 2a, b, 3a). Several large loops of UNC79–UNC80 were unresolved, probably due to their intrinsic flexibility, as indicated in the predicted structures (Fig. 2c; Supplementary Fig. S5a). The super-helical scaffold of UNC79–UNC80 is reminiscent of the largest known ion channel RyR, whose cytosolic super-helical domains provide a huge platform for the binding of multiple modulators^22^ (Fig. 2d). The large assembly of UNC79–UNC80 could allow it to play a similar role in recruiting regulators of the channelosome, such as Src, a member of the SFKs that can phosphorylate NALCN, UNC79, and UNC80 to activate the channelosome^21^. Except for the HA repeats, UNC79 and UNC80 both contain an additional C-terminal helix (Supplementary Fig. S5b). UNC80 also adopts an extra small domain between the two helices of HA18 repeat (Supplementary Fig. S5c). Structural comparison analysis by the DALI server indicates that this domain shares the same fold as FERM domain, a widespread protein module involved in membrane trafficking of proteins^23^. This structural observation is consistent with the functional role of the UNC79–UNC80 assembly in modulating the neuronal localization and/or stabilization of the NALCN channelosome^7,9^.

UNC79 and UNC80 form extensive interactions through three interfaces: the N-interface (UNC79-N and UNC80-C), the M-interface (middle region of both UNC79 and UNC80), and the C-interface (UNC79-C and UNC80-N), with a total interface area of about 5000 Å^2^ (Fig. 3a–d; Supplementary Fig. S5b). The sequences of these interacting regions are largely conserved (not shown), suggesting a shared UNC79–UNC80 assembly mechanism among different species. Electrophysiological experiments in HEK293T system show segment deletions of UNC79 or UNC80 in both the N-interface and the C-interface significantly reduce the NALCN conducting currents to background levels (Fig. 3e; Supplementary Fig. S1). We identified a pair of critical residues in the N-interface, UNC79–Y264 and UNC80–R2844, that likely form cation–π interaction (Fig. 3d). Single mutation of UNC79–Y264R or UNC80–R2844E results in a reduction of the NALCN-mediated currents to less than one-third of wild type (WT), while the double mutations could rescue the currents close to WT level (Fig. 3e; Supplementary Fig. S1). These results are also supported by a previous study, which showed the interaction between the C-terminus of UNC80 and UNC79 (N-interface) is indispensable for achieving dendritic localization of the channelosome^9^. The M-interface forms the largest contact area, involving HA13–19 repeats of UNC79 and HA15–22 repeats of UNC80. A single mutation at the M-interface, UNC80–K2152E, leads to a reduction in current to approximately half of WT (Fig. 2e; Supplementary Fig. S2). Together, our results indicate that all three interfaces are critical for UNC79–UNC80 assembly and the channelosome function.

### Interactions between NALCN and the UNC79–UNC80 assembly

**Fig. 4:**
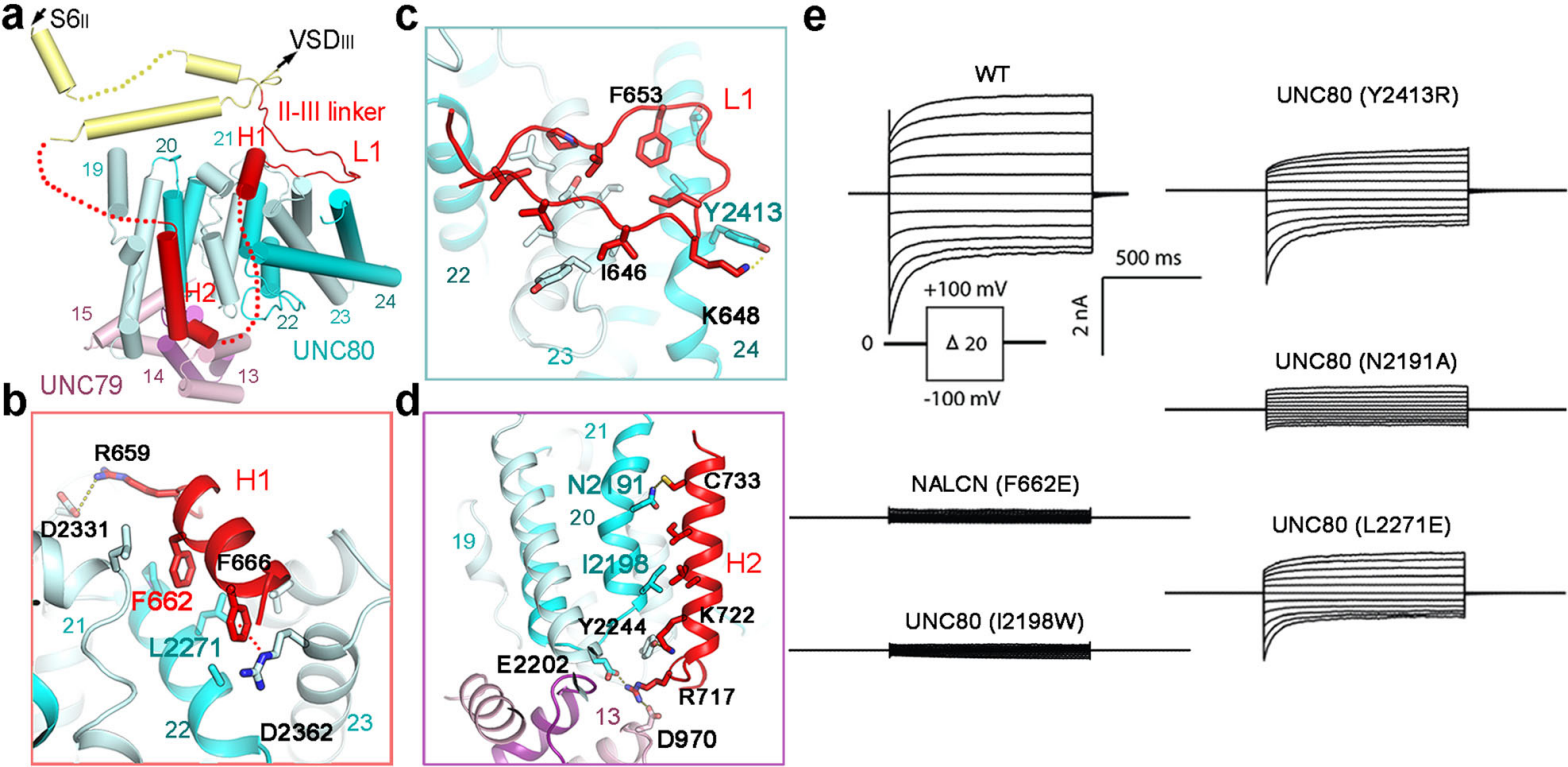
Interaction between NALCN II–III linker and the UNC79–UNC80 assembly. **a** Interaction interface between the II–III linker of NALCN and the UNC79–UNC80 assembly. Flexible regions that are invisible and unmodelled in the structure are indicated by dashed lines. **b**–**d** Close-up views of the interfaces between II–III linker of NALCN and the UNC79–UNC80 assembly. Key residues that are involved in interface interactions are shown as sticks. The residues that are mutated and tested by electrophysiological experiments are labeled in color. **e** Representative current traces from HEK293T cells expressing the WT NALCN channelosome and the constructs with indicated mutations on the interfaces between the II–III linker of NALCN and the UNC79–UNC80 assembly.

The II–III linker of NALCN mediates the association between the NALCN–FAM155A subcomplex and the UNC79–UNC80 assembly (Figs. 2a, 4a). Notably, these interacting segments of II–III linker (Pro638–Arg669, Arg715–Arg737) were unresolved in the previous NALCN–FAM155A subcomplex structure and was only observed when UNC79 and UNC80 were associated (Supplementary Fig. S6a, b). The interacting segments of the II–III linker mainly includes a loop (L1) and two helices (H1 and H2) (Fig. 4a; Supplementary Fig. S3c). These segments form three specific interaction interfaces with adjacent residues from UNC79 and UNC80, providing the molecular basis for the formation of the intact NALCN channelosome (Fig. 4b–d). Single mutation of NALCN in the interacting interface, F662E, dramatically reduces the currents to background levels (Fig. 4e; Supplementary Fig. S1c, f). The expression level and cellular localization of this NALCN mutant were indistinguishable from WT, suggesting that the changes of currents were due to disruption of the interface (Supplementary Fig. S1a, b). Single mutations of the residues on UNC80 from all three interfaces (N2191A, I2198W, L2271E, and Y2413R) that interact with the II–III linker of NALCN also reduce the NALCN-mediated currents to varying degrees, suggesting the importance of II–III linker interfaces in the assembly and function of the channelosome (Fig. 4e; Supplementary Fig. S1e, f). Notably, the key interacting residues on both NALCN and UNCs are mostly invariant across vertebrate and invertebrate species, indicating that the assembly mechanism of NALCN channelosome are evolutionarily conserved (Supplementary Fig. S6c–e). The absence of a conserved II–III linker in other 4 × 6 TM channels rationalizes the specificity of the UNC79–UNC80 assembly for NALCN.

### Structural mapping of pathogenic mutations in UNC80

**Fig. 5:**
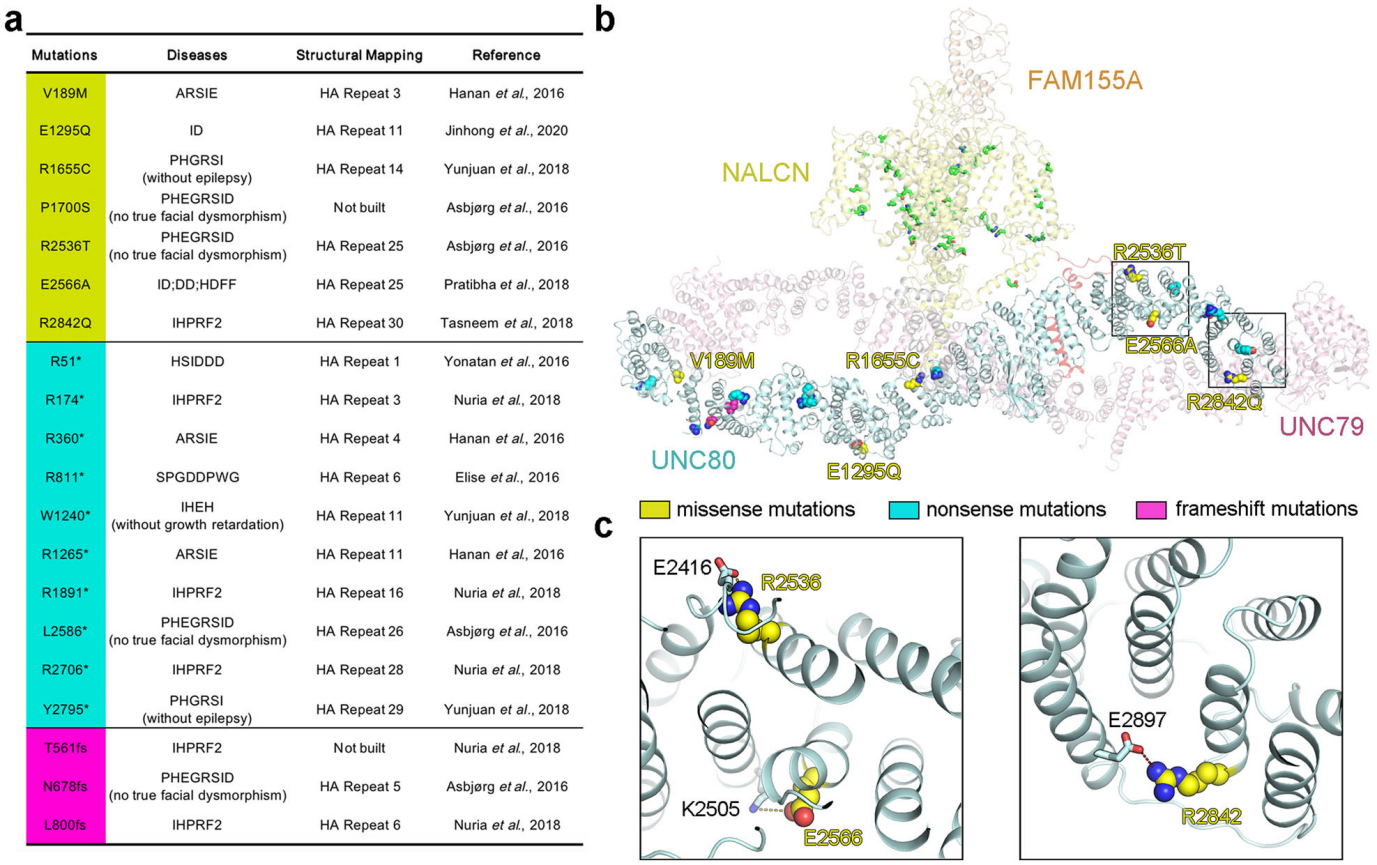
Structural mapping of disease-related mutations. **a** A list of previously reported disease-related mutations in UNC80^9,11,37–43^. The missense mutations, nonsense mutations, and frameshift mutations are shaded in yellow, cyan, and magenta, respectively. ARSIE, autosomal-recessive severe infantile encephalopathy; ID, intellectual disability; IHPRF2, Infantile hypotonia with psychomotor retardation and characteristic facies-2; PHGRSI, persistent hypotonia, growth retardation and self-injury; PHEGRSID, persistent hypotonia, encephalopathy, growth retardation, and severe intellectual disability; HSIDDD, hypotonia, severe intellectual disability, dyskinesia and dysmorphism; SPGDDPWG, spastic paraplegia, global developmental delay and poor weight gain; IHEH, infantile hypotonia, epilepsy and hyperactivity; HDFF, hypotonia and dysmorphic facial features. **b** Structural mapping of disease-related mutations onto the NALCN channelosome. The diseased-related mutations in NALCN (shown in green sticks) have been discussed in our previous study^17^. Disease-related mutation sites in UNC80 are shown as spheres and colored by mutation type. Close-up views of the boxed region are shown in **c**. **c** Enlarged views of three missense mutation sites in UNC80. Hydrogen bonds are indicated by dashed lines. The mutations are likely to break local hydrogen bonds to affect UNC80 folding and local stability.

The structure of the intact channelosome provides a template to map the disease-related mutations for interpretation of their potential pathogenic mechanisms. Most of the pathogenic mutations are located in NALCN and UNC80 (Fig. 5). Our previous study has revealed that the mutations in NALCN mainly focus on the pore regions that may directly affect the ion permeation properties^17^. In this study, we have summarized the reported disease-related mutations in UNC80 and found that they are sporadically distributed (Fig. 5a, b). According to the structural and electrophysiological results, nonsense mutations and frameshift mutations of UNC80 could affect the channelosome function through disruption of the UNC79–UNC80 assembly in the N-interface and/or M-interface (Fig. 5b). The missense mutations of UNC80 may affect the channelosome function by interfering with the local folding and stability of UNC80. For instance, the missense mutations sites R2536, E2566, and R2842 all form hydrogen bonds with residues from the adjacent HA repeat (Fig. 5c). It is to be noted that mutations on the inter-subunit interfaces were rarely reported, probably because mutations in these regions are neonatal lethal, as reported in the phenotype of UNC80 knock-out mice^9^.

## Discussion

Our study reports an overall architecture of the human NALCN channelosome, providing an important framework to understand its assembly, regulation, and function. The structure has revealed the interfaces between UNC79 and UNC80, and between the II–III linker of NALCN and the UNC79–UNC80 assembly, which are critical for the formation of intact channelosome and its function. Apart from the structural evidence, results from our patch clamp approach show that the presence of UNC79–UNC80 assembly in the channelosome is essential for the electrophysiological function, which are consistent with other previous studies^9,18,20^ (Fig. 1c).

The unique intracellular super-helical scaffold of the channelosome provides a molecular basis for the potential coupling between its conformational change and the gating of NALCN, as reported in RyR1^24^. We speculate that the intracellular scaffold not only plays an essential role in directly mediating the channelosome function, but also establishes a platform for the binding of a variety of other regulators, as observed for the bound CaM. Some other proteins, including Src^21^ and M3R^25^, have been reported to regulate the channelosome activity through physical interactions. It is likely that these potential regulators modulate the channelosome property through interacting with the channelosome intracellular scaffold. However, structural evidence for the interaction of Src, M3R, and other potential regulators with the channelsome needs further studies.

During the preparation of this manuscript, a similar work was reported elsewhere^26^, and the major structural observations and conclusions are mostly consistent with our work. There are differences in the protein sample preparation strategies, electrophysiological designs, and some structural interpretations, which make the two works complement each other. The C-interface, considered to be dispensable in that work, turns out to be important for channelosome function in our study. This discrepancy may be due to different electrophysiological systems used in the two works. An extra interface between the I–II linker of NALCN and UNC79 C-half identified in that work was not observed in our study, probably due to different sample conditions (Supplementary Fig. S7). It seems that I–II linker is not as essential as the II–III linker in association between NALCN and the UNC79–UNC80 subcomplex, as alterations of the I–II linker did not obviously impact the channelosome function^26^. The absence of the I–II linker interacting interface in our study suggests that it is highly dynamic, which in turn leads to a more pronounced local conformational flexibility (Supplementary Video S1). Combined with the other study^26^, our structure suggests an asynchronous assembly process between NALCN–FAM155A subcomplex and the UNC79–UNC80 assembly, in which the formation of the I–II linker interface may be a subsequent step after the formation of II–III linker interface to further stabilize the channelosome.

## Materials and methods

### Expression and purification of the human NALCN channelosome

The codon optimized DNAs of human FAM155A, NALCN, UNC80 and UNC79 were individually cloned into a pCAG vector behind a CMV promoter. FAM155A and UNC80 were untagged. The carboxyl termini of NALCN and UNC79 were fused with a green fluorescent protein (GFP) and a 2× FLAG tag, respectively. The four plasmids were co-transfected at a molar ratio of 2:3:3:10 into HEK293F cells (Invitrogen) with polyethylenimine (PEI) when the cell density reached 2 × 10^6^ cells/mL. Transfected cells were collected after 48 h culturing in SMM 293-TII medium (Sino Biological Inc.) under 5% CO_2_ at 37 °C. Collected cells were resuspended in buffer containing 25 mM MOPS (pH 7.4), 300 mM NaCl, and protease inhibitor cocktail including 2 mM phenylmethylsulfonyl fluoride (PMSF), 1.3 μg/mL aprotinin, 0.7 μg/mL pepstatin, and 5 μg/mL leupeptin, and subsequently lysed by sonication. Subsequently, 1% (w/v) lauryl maltose neopentyl glycol (LMNG, Anatrace), 0.12% (w/v) cholesteryl hemisuccinate Tris salt (CHS, Anatrace) and 0.6 mg/mL GFP-nanobodies were added and incubated at 4 °C for 2 h. The insoluble fraction was precipitated by ultracentrifugation at 255,700× *g* for 40 min, and the supernatant was applied to glutathione sepharose 4B resin (GS4B, GE Healthcare). The protein-bound resin was washed with 25 mM MOPS (pH 7.4), 400 mM NaCl, and 0.015% GDN (Anatrace). The target protein was eluted with buffer containing 15 mM Tris–HCl (pH 8.0), 15 mM Tris–HCl (pH 8.8), 150 mM NaCl, 0.015% GDN, and 13 mM reduced glutathione. The eluent was then applied to anti-FLAG G1 affinity resin (GenScript) and incubated with the resin for 1 h at 4 °C. The resin was washed with buffer containing 25 mM MOPS (pH 7.4), 150 mM NaCl, and 0.015% GDN. The protein was then eluted with buffer containing 25 mM MOPS (pH 7.4), 150 mM NaCl, 0.015% GDN and 250 μg/mL FLAG peptide. The eluent was concentrated and cross-linked using 2 mM bis(sulfosuccinimidyl) suberate (BS^3^) at 4 °C for 1.5 h and then quenched by 25 mM Tris–HCl (pH 8.0). The sample was then applied to size-exclusion chromatography (Superose 6, 10/30, GE Healthcare) in buffer containing 25 mM MOPS (pH 7.4), 150 mM NaCl and 0.015% GDN. The fractions containing the NALCN-FAM155A–UNC79–UNC80 complex were pooled and concentrated to about 0.4 mg/mL for electron microscopy and mass spectrometric analysis.

### Cryo-EM sample preparation and data collection

For cryo-EM sample preparation, aliquots (4 μL) of the protein sample were loaded onto glow-discharged grids (Quantifoil R 1.2/1.3 Cu 300 mesh) coated with 2 nm carbon film. Under 100% humidity at 8 °C, the grids were blotted for 3 s with Vitrobot (Mark IV, Thermo Fisher Scientific) after waiting for 60 s, and immersed in liquid ethane cooled by liquid nitrogen. The imaging system comprises of a Titan Krios operating at 300 kV, a Gatan K3 Summit detector, and a GIF Quantum energy filter with a 20-eV slit width. Movie stacks were automatically collected via EPU (Thermo Fisher Scientific) in super-resolution mode (×81,000 magnification), with a defocus range from −1.4 to −2.0 μm. Each image stack was exposed for 2.56 s with 0.08 s per frame, resulting in 32 frames and ~50 e^−^/Å^2^ of total dose.

### Cryo-EM data processing

A simplified data processing flowchart can be found in Supplementary Fig. S2f. 33,028 movie stacks (1.087 Å/pixel) were collected and motion-corrected using MotionCor2^27^. The following data processing procedures were performed using CryoSPARC v3^28^. After patch-CTF estimation, around six million particles were automatically picked with picking templates generated from template-free 2D classification. Particles were extracted with a box size of 512 pixels. After two rounds of 2D classification, a total of 590,796 particles were selected from good 2D classes. An initial low-resolution map was generated using these particles as the reference for the subsequent non-uniform refinement job^29^, yielding a reconstruction with identifiable secondary structure features in the large cytosolic region. The conformational dynamics of the particles were analyzed by the 3D Variability Analysis job^30^ in CryoSPARC.

To improve the overall map quality, a heterogeneous refinement (*K* = 5) was performed and resulted in a class with better secondary structure feature in both the cytosolic and transmembrane regions, consisting of 174,294 selected particles. Further non-uniform refinement of these selected particles yielded a reconstruction of the whole complex at a resolution of 4.5 Å (Map1). To further improve the resolution of local regions, several different masks were applied for local refinements of corresponding re-centered particles. Basically, 383,924 good particles selected from an extra round of 2D classification were first recentered focusing on a masked region. The re-extracted particles were then subtracted using the given mask and applied for local refinement. We have tried masks on NALCN–FAM155A, UNC79–UNC80, UNC79 N-half + UNC80 C-half, and UNC79 C-half + UNC80 N-half. One of such attempts significantly improved the local resolution to 3.3 Å, when the mask surrounding UNC79 N-half + UNC80 C-half was applied (Map2). However, other attempts did not generate maps with significantly improved quality compared to Map1 and therefore were not used. Map resolutions were determined by gold-standard Fourier shell correlation (FSC) at 0.143.

### Model building and refinement

A composite map combining Map1 and Map2 was generated for model building. The structure of NALCN–FAM155A (PDB: 7CM3), CaM (PDB: 6MUD) and the predicted structures of UNC79 and UNC80 segments by AlphaFold2^31^ were used as the initial templates. The template models were first docked into the cryo-EM map in Chimera^32^ and then manually adjusted in Coot^33^. Large flexible linkers of the UNC79 and UNC80 that were invisible in the density map were removed. The modeling of N-interface and C-interface between UNC79 and UNC80 were facilitated by predicated complex structures by AlphaFold-Multimer^34^. The modeling of CaM was guided by the cross-linking mass spectrometry data. The map quality of Map2 is relatively high that allows for side chain assignment for some local regions, including the L1, H1, and H2 segments of the NALCN II–III linker. The final model was refined against the composite map by PHENIX^35^ in real space (phenix.real_space_refine) using the rigid-body parameter. The final overall model was validated using phenix.validation_cryoem. The statistics of the 3D reconstructions and model refinement, and a summary of the overall model can be found in Supplementary Tables S1 and S2, respectively.

### Western blotting assay

HEK293F cells were transfected with same amounts of plasmids of C-terminal GFP tagged NALCN (WT/mutant)–FAM155A–UNC79–UNC80 in the same ratio for protein expression. Cells were lysed 48 h post transfection in RIPA buffer (Solarbio) with 1.3 μg/mL aprotinin, 0.7 μg/mL pepstatin, 5 μg/mL leupeptin, and 2 mM phenylmethylsulfonyl fluoride (PMSF), followed by sonication. Lysates normalized by the expression level of GAPDH were loaded onto 14% sodium dodecyl sulfate–polyacrylamide gels for electrophoresis (SDS–PAGE). The separated proteins were then transferred to poly-vinylidene fluoride (PVDF) membranes (Merck Millipore). After blocking with 3% BSA for 1 h at 37 °C in Tris-buffered saline with Tween-20 (TBS-T), the membranes were incubated with the primary antibodies for 1 h at 37 °C and then with the horseradish peroxidase (HRP) labeled secondary antibody (1:5000 dilution, CWBIO) for 1 h. WB bands were visualized with the eECL western blot kit (CWBIO). The primary antibodies used were anti-GFP (1:3000 dilution, Abmart) and anti-GAPDH (1:5000 dilution, Proteintech).

### HEK-293T cell culture and transfection

HEK293T cells authenticated by short tandem repeat DNA profiling (gifted from Dr. Chunqing Song) were grown in SMM 293-TII medium (Sino Biological Inc.) supplemented with 10% fetal bovine serum (Biochannel) and 1% penicillin–streptomycin (10,000 U/mL; Cytiva) at 37 °C in a 5% CO_2_ humidified growth incubator. Cells between passages 10 and 20 were used for electrophysiological experiments. Here, 20–24 h before transfection, HEK293 cells were detached by treatment with 0.25% trypsin for 5 min at room temperature and seeded into new 12-well culture plates at 50% confluency. For patch-clamp experiments, cells reaching 60%–70% confluency were transiently transfected with 3.5 μg total expression plasmids using PEI. All plasmids used for transfection were constructed into the same vector for protein expression. For the WT NALCN channelosome, NALCN_GFP_, FAM155A, UNC79_FLAG_, and UNC80 plasmids were mixed at the molar ratio of 3:2:10:3, same to the ratio for protein expression. The different mutation or deletion constructs of NALCN, UNC79, UNC80 contain the same tag to the WT. For each mutant group, the corresponding WT plasmid was replaced by the mutant and other three plasmids are unchanged with the exact same ratio. The transfection mix was removed after 6–8 h, and cells were washed with PBS and cultured in supplemented DMEM. For mock-transfected cells, the vector expressing GFP was used. For NALCN alone-transfected cells, 2.2 μg total plasmids of NALCN_GFP_ was used.

### Immunostaining and imaging

About 8–10 h before staining, the transfected HEK293T were seeded on poly-d-lysine-coated glass coverslips. Samples were rinsed with 1× phosphate-buffered saline (PBS) once, and then fixed in 4% paraformaldehyde (pH 7.4) for 10 min at room temperature, followed by permeabilization with 0.25% Triton-X100 (Sigma) in PBS for 10 min at room temperature. Samples were blocked for 1 h in 5% bovine serum albumin (BSA) in PBS and incubated with rabbit anti-GFP antibody (1:500; Abcam) in 5% BSA overnight at 4 °C. After rinsed with PBS, samples were incubated with Alexa 488 secondary antibody (1:1000, Life Technologies) in 5% BSA for 1 h at room temperature and stained with 4′,6-diamidino-2-phenylindole DAPI (Lablead) for 5 min at room temperature. Images were captured by the FV3000-IX83 confocal system (Olympus).

### Whole-cell patch clamp electrophysiology

Electrophysiological experiments were conducted 72–96 h after transfection and the transfected HEK-293T cells were seeded on new poly-d-lysine-coated glass coverslips at least 1 h before recording. Patch clamp recordings were performed with a HEKA EPC10 amplifier with PatchMaster software (HEKA) in whole-cell configuration at room temperature (23 ± 2 °C). Micropipettes were pulled with a P-1000 flaming/Brown Micropipette Puller System (Sutter Instrument) and fire-polished with Micro Forge MF2 (Narishige) from the 1.5/1.2 mm (outer diameter (OD)/inner diameter (ID)), thin-walled glass (Sutter Instrument). The series resistance of micropipettes was typically 3–5 MΩ. The recording micropipettes were filled with internal solution containing: 136 mM NaCl, 5 mM EGTA, 10 mM HEPES, and 2 mM Na_2_ATP (adenosine 5′-triphosphate) (pH 7.2) with NaOH. The recipe of external solution was: 150 mM NaCl, 10 mM HEPES, and 30 mM d-(+)-glucose (pH 7.4) with NaOH. Solution osmolarity was ~290–310 mOsm/L adjusted with glucose, and ~5 mOsm/L lower in the internal solutions than the external solutions of the same experiment.

Traces were acquired at a repetition interval of 4 s. Currents signals were sampled at 25 kHz and filtered at 1 kHz. The holding potential was 0 mV. *I*–*V* curves were generated from a group of step potentials (−100 to +100 mV with a 20 mV increment) and at the steady-state during the last 20 ms of voltage steps was averaged. Data from patch clamp recordings were analyzed in Igor Pro (WaveMatrix) and Graphpad Prism. Statistically significant differences (*P* < 0.05) between means of two groups were determined by a two-tailed *t*-test. Data are presented as means ± SEM or mean ± SD.

### Cross-linking mass spectrometry (XL-MS) analysis

The peak fractions after gel filtration purification containing the BS^3^ cross-linked protein sample were applied to SDS–PAGE gel and stained with Coomassie Blue G-250. The cross-linked band containing the NALCN channelosome was cut into pieces and put in 50 mM ammonium bicarbonate with trypsin at 37 °C overnight for prior reduction and alkylation. The digested products were extracted twice with 1% formic acid in 50% acetonitrile aqueous solution and dried to reduce volume by speedvac.

For LC–MS/MS analysis, the peptides were separated by a 65 min gradient elution at a flow rate 0.300 µl/min with the Thermo EASY-nLC1200 integrated nano-HPLC system, which is directly interfaced with the Thermo Q Exactive HF-X mass spectrometer. The analytical column was a home-made fused silica capillary column (75 µm ID, 150 mm length; Upchurch, Oak Harbor, WA) packed with C-18 resin (300 A, 3 µm, Varian, Lexington, MA). Mobile phase A consisted of 0.1% formic acid, and mobile phase B consisted of 80% acetonitrile and 0.1% formic acid. The mass spectrometer was operated in the data-dependent acquisition mode using the Xcalibur 4.1 software and there is a single full-scan mass spectrum in the Orbitrap (350–1800*m*/*z*, 60,000 resolution) followed by 20 data-dependent MS/MS scans at 30% normalized collision energy. Each mass spectrum was analyzed using the Proteome Discoverer 2.4 and pLink 2.3.9^36^ for the database searching and cross-linking analysis.

## Supplementary information

Supplementary Figures and Tables

Supplementary Video S1

## Data Availability

Coordinates and corresponding EM maps of the human NALCN channelosome (PDB: 7WJI; EMDB: EMD-32544) have been deposited in the Protein Data Bank (http://www.rcsb.org) and the Electron Microscopy Data Bank (https://www.ebi.ac.uk/pdbe/emdb/), respectively. Source data and other related materials are available from the corresponding authors upon reasonable request.
