# Supplementary material for: Architecture of the human NALCN channelosome
Source: Cell Discov. 2022 Apr 6;8:33. doi: 10.1038/s41421-022-00392-4 (PMC8986805; doi:10.1038/s41421-022-00392-4)
Supplement: Supplementary file 1 — Supplementary Figures and Tables [file 41421_2022_392_MOESM1_ESM.pdf]

Supplementary Information for

Architecture of the human NALCN channelosome

Lunni Zhou, Haobin Liu, Qingqing Zhao, Jianping Wu*, and Zhen Yan*

*To whom correspondence should be addressed: Z. Yan (yanzhen@westlake.edu.cn) or J. Wu (wujianping@westlake.edu.cn)

This file includes:

Supplementary Figs. S1-S7
Supplementary Tables S1-S2
Supplementary Video title

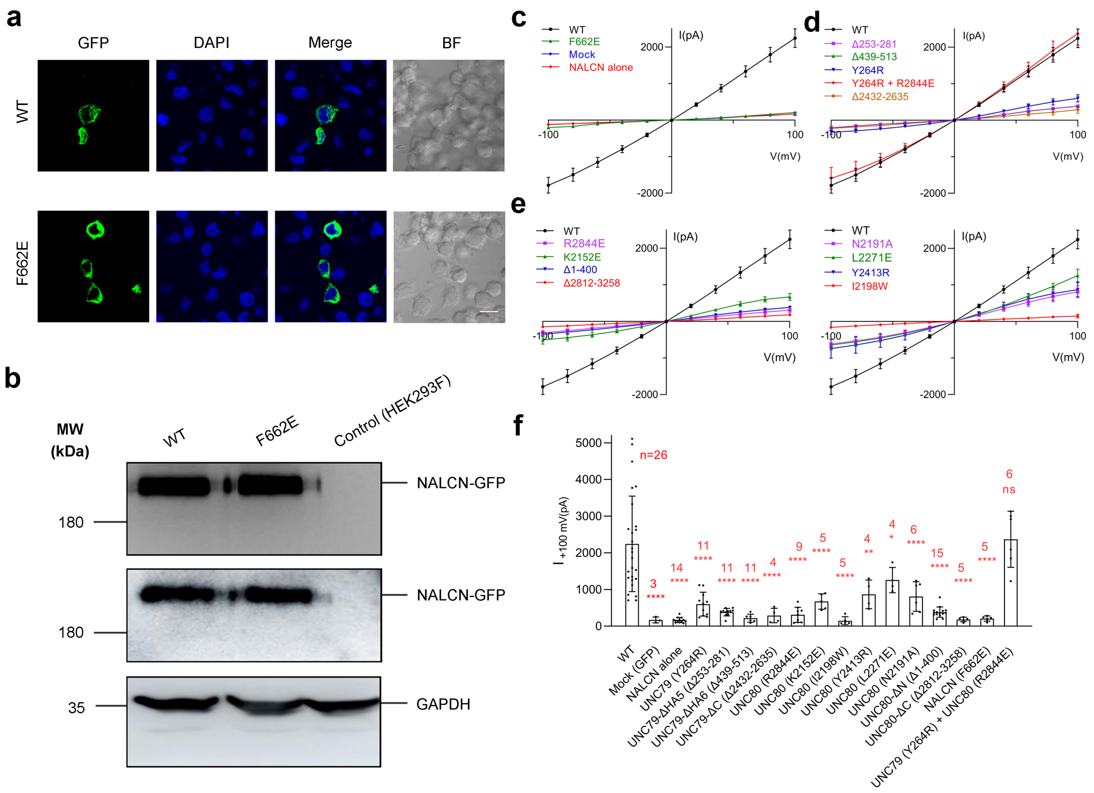

Supplementary Fig. S1. Expression and electrophysiological characterizations of the NALCN channelosome by whole-cell patch clamp recordings. (**a**) NALCN mutant F662E shares similar cellular localization pattern to WT in HEK293T cells. Images were recorded in HEK293T cells transfected with FAM155A, UNC79, UNC80, and NALCN-WT/F662E (with C-terminal GFP tag), respectively. $NALCN_{GFP}$-WT and mutant were stained with GFP antibody (green). Cell nuclei were stained with DAPI (blue). Scale bar: 10µm. **(b)** NALCN mutant F662E has similar expression level to WT in HEK293F cells. Upper: expression levels of NALCN detected by in-gel GFP fluorescence; middle: expression levels of NALCN detected by western blotting using anti-GFP antibody; lower: expression of control protein detected by anti-GAPDH antibody. **(c)** Steady-state current-voltage (I-V) curves for HEK293T cells transiently expressing the WT NALCN channelosome, NALCN mutant F662E, Mock and NALCN alone. All error bars in **c-e** represent s.e.m. **(d)** I-V curves for the WT NALCN channelosome, and four mutations of UNC79 including Δ253-281, Δ439-513, Δ2432-2635 (ΔC), and Y264R. "Y264R + R2844E" means both the WT UNC79 and UNC80 were replaced with the indicating mutation constructs. **(e)** I-V curves for the WT NALCN channelosome, and eight mutations of UNC80 including R2844E, K2152E, Δ1-400 (ΔN), Δ2812-3258 (ΔC), N2191A, L2271E, Y2413R, and I2198W. **(f)** Steady-state currents at +100mV of all experimental groups presented in this study. N number is labelled above each column. ns P > 0.05, * P≤ 0.05, ** P ≤ 0.01, *** P ≤ 0.001, **** P ≤ 0.0001. A two-tailed unpaired t-test was used. All error bars in this panel represent s.d.

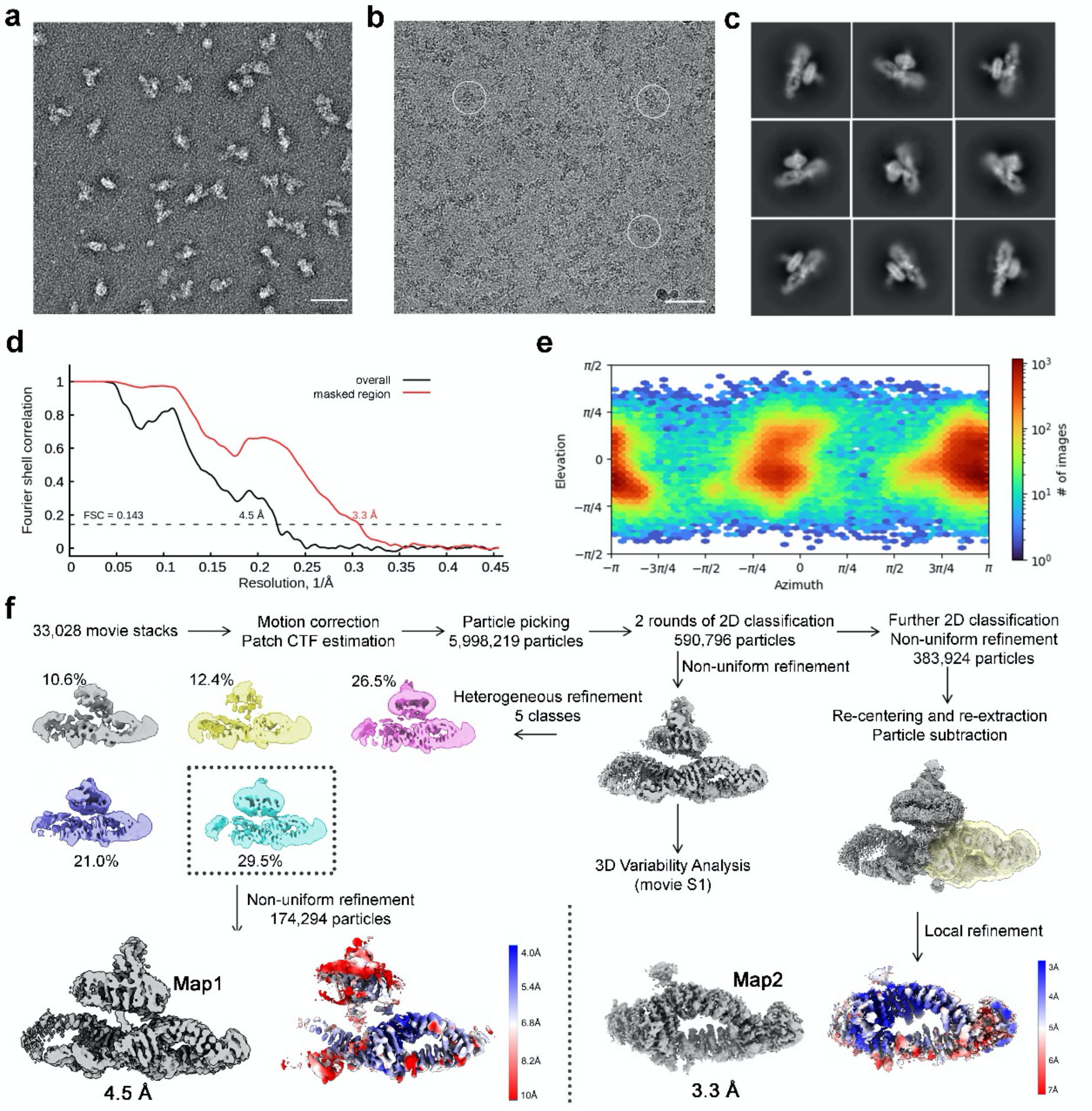

Supplementary Fig. S2. Cryo-EM analysis of the human NALCN channelosome. **(a)** Negative staining image of the NALCN channelosome. The NALCN channelosome particles display a characteristic triangle shape. Scale bar: 50 nm. **(b)** Representative cryo-EM micrograph of the NALCN channelosome. Selected particles are indicated by white circles. Scale bar: 50 nm. **(c)** Representative two-dimensional (2D) class averages of the NALCN channelosome. **(d)** Gold standard FSC curves for the 3D reconstructions. The FSC curves for the reconstructions of the overall map (Map1) and the local map containing the UNC80 C-half and the UNC79 N-half regions (Map2) are shown in black and red, respectively. **(e)** Angular distribution of the particles of the final overall reconstruction generated by cryoSPARC[1]. **(f)** A simplified data processing workflow of the cryo-EM dataset. Local resolution maps were estimated by cryoSPARC and generated in Chimera[2].

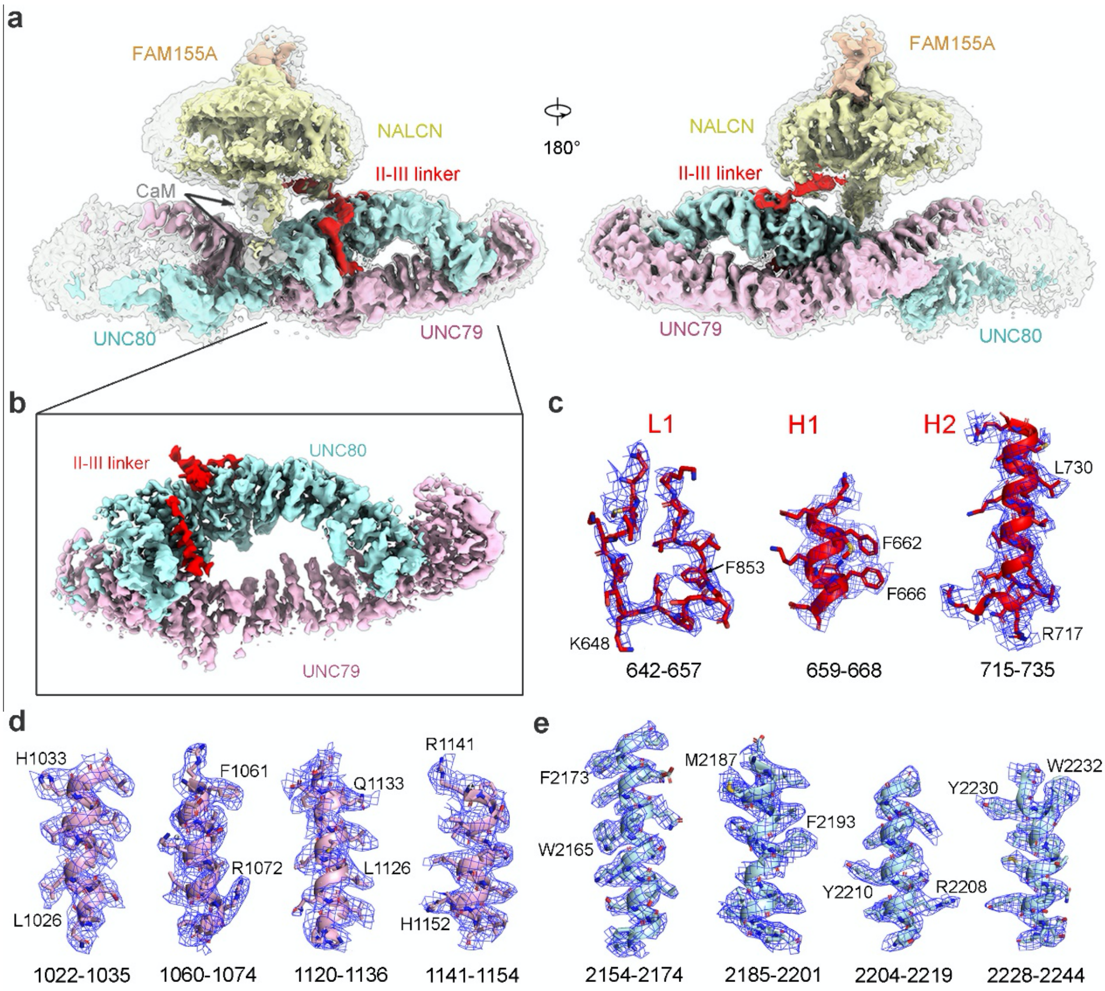

Supplementary Fig. S3. EM density maps of the human NALCN channelosome. **(a)** The overall EM density map of the NALCN channelosome. The map contoured at 0.35 in ChimeraX[3] is colored by components: NALCN (pale-yellow), FAM155A (wheat), UNC80 (light blue), UNC79 (light pink), CaM (gray). The II-III linker of NALCN is highlighted in red. The detergent micelle in the transmembrane region and the full cytosolic region were outlined by the same map contoured at 0.25 in transparency. **(b)** The local map with improved density quality. This region includes the UNC80 C-half, UNC79 N-half, and the NALCN II-III linker. The color scheme is the same as in **(a)**. **(c-e)** Electron density maps of segments from **(c)** NALCN II-III linker, **(d)** UNC79, and **(e)** UNC80. The maps shown as blue meshes are contoured at 5 σ in PyMOL[4].

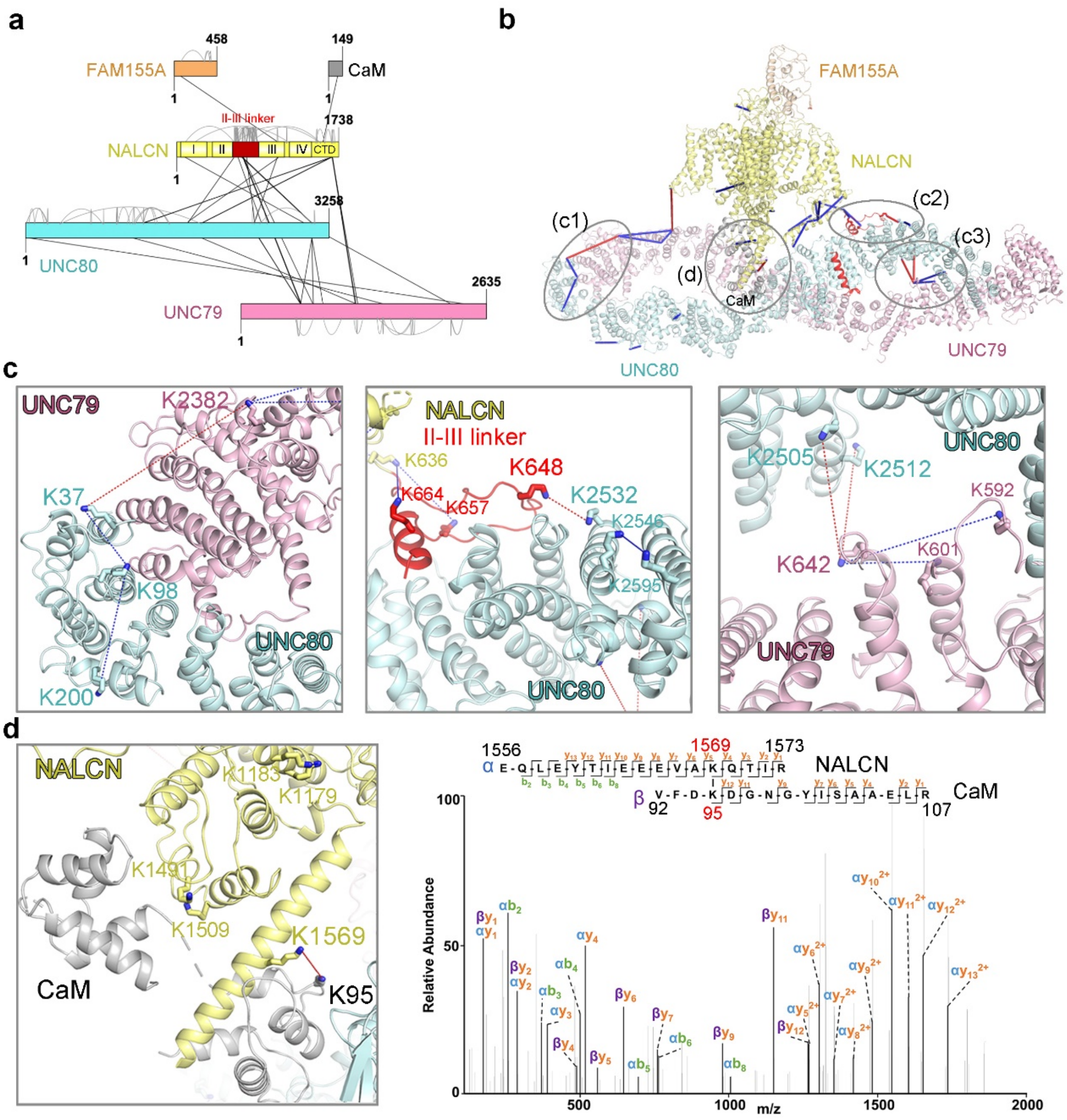

Supplementary Fig. S4. Structural validation by XL-MS analysis. (a) BS^3 crosslinks illustrated on the schematic diagrams of the NALCN channelosome components. Inter-protein cross-links are shown in black and intra-protein links in gray. Within the primary structure of NALCN, the four homologous repeats (I-IV) and the C-terminal domain (CTD) of NALCN are indicated. **(b)** Structural mapping of the cross-links. Most of the cross-links are within a distance of 25 Å in the structure. Inter-subunit and intra-subunit cross-links are colored in red and blue, respectively. Close-up views of the circled region are shown in **c-d**. **(c)** Zoom-in views of the inter-subunit cross-links in the C-interface between UNC79 and UNC80, the interface between II-III linker of NALCN and UNC80, and a region near the N-interface between UNC80 and UNC79. **(d)** Structural mapping of a cross-link between NALCN-K1569 and CaM-K95, suggesting physical interaction between the CTD of NALCN and the C-lobe of CaM. The MS spectra of this cross-link is shown on the right.

a

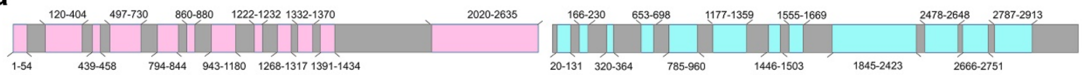

b

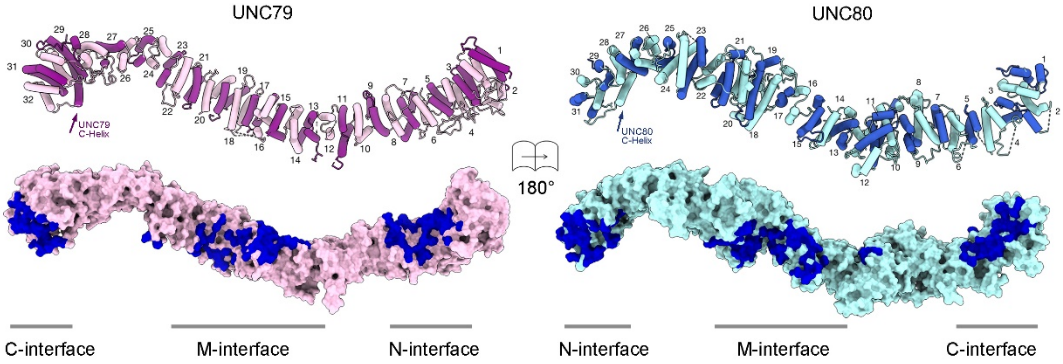

c

No.	Chain	Z-score	rmsd	lali	nres	%identity	Description
1	3qij-B	11.5	4.5	91	273	11	MOLECULE: PROTEIN 4.1 (FERM DOMAIN)
2	2d10-D	10.8	4.0	88	297	8	MOLECULE: RADIXIN
3	2d10-B	10.8	3.9	88	297	8	MOLECULE: RADIXIN
4	1ef1-B	10.8	4.5	90	294	7	MOLECULE: MOESIN
5	6fnn-D	10.8	1.8	73	90	10	MOLECULE: 26S PROTEASOME REGULATORY SUBUNIT N11-LIKE PROTEIN
6	6d21-A	10.8	3.0	86	280	10	MOLECULE: FERM, RHOGEF AND PLECKSTRIN DOMAIN PROTEIN 2
7	4rm8-B	10.6	4.2	89	367	7	MOLECULE: EZRIN
8	7lwh-A	10.5	3.9	88	315	7	MOLECULE: MERLIN
9	3bin-A	10.5	4.7	91	281	7	MOLECULE: BAND 4.1-LIKE PROTEIN 3 (FERM DOMAIN)
10	4rm9-A	10.5	4.0	88	368	8	MOLECULE: EZRIN

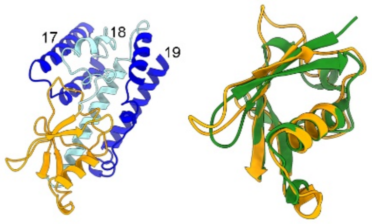

Supplementary Fig. S5. Structural details of UNC79 and UNC80. (a) Schematic diagrams showing the modelled region (in color) and unmodelled region (in gray) of UNC79 and UNC80. The unmodelled region are mostly flexible linkers. **(b)** Super-helical structure of UNC79 and UNC80. The number of the HA repeats and the C-terminal helix (C-Helix) are labelled. The C-Helix of UNC79 and UNC80 attaches to their fourth to last HA repeats. The lower surface presentations indicate the three interfaces (highlighted in blue) between UNC79 and UNC80. **(c)** DALI analyses of the structural homologs of the FERM-like domain (1995-2078aa) of UNC80. Top 10 existing structures showing high structural similarities are listed in the left table. The right panel shows cartoon presentations of the FERM-like domain and HA17-19 repeats of UNC80; and structural overlay of UNC80 (1995-2078) (in yellow) with FERM domain (PDB: 6D21) (in green).

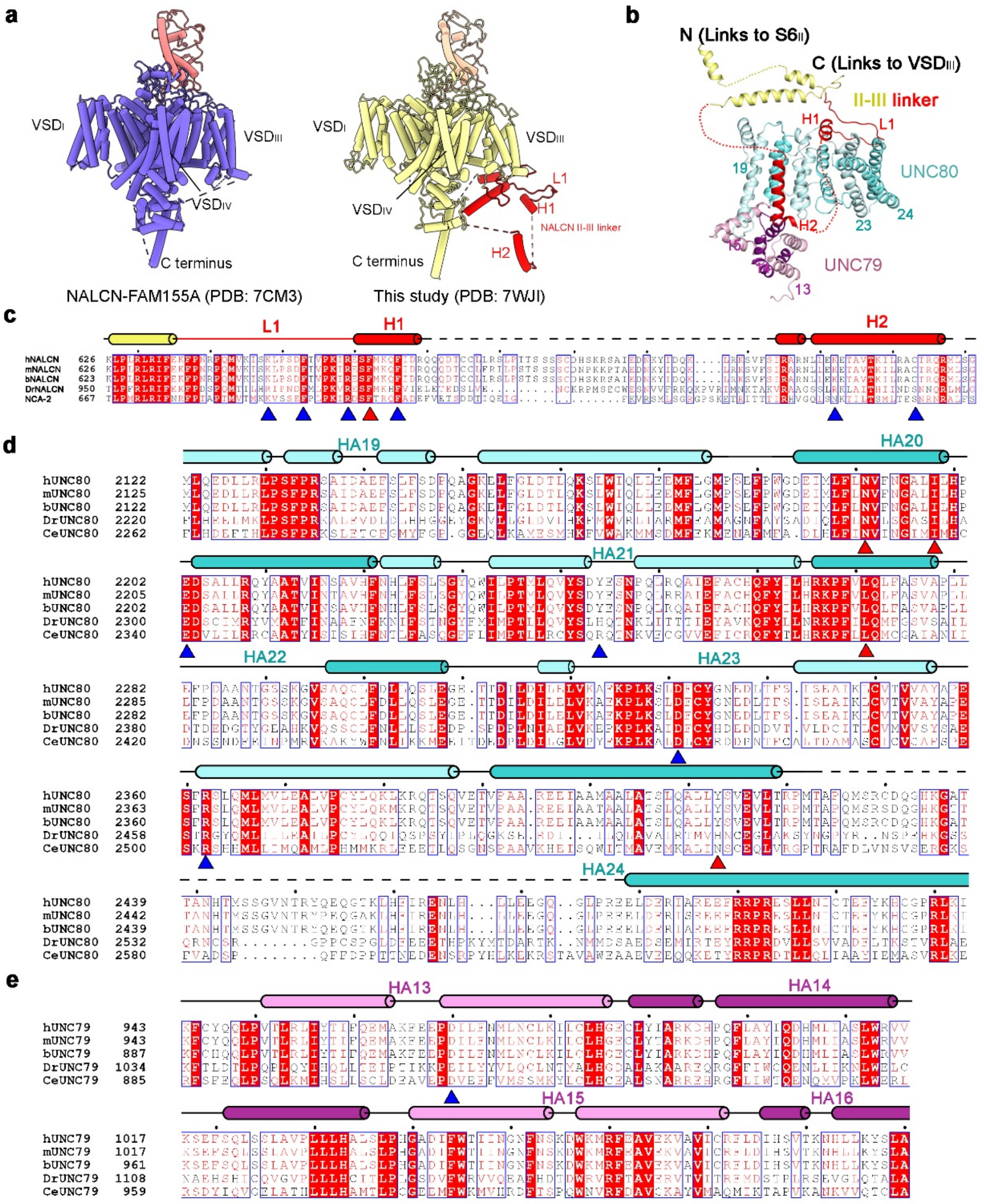

Supplementary Fig. S6. Structure and sequence alignments of interface segments between NALCN and UNCs. (a) Structural comparison of the NALCN-FAM155A subcomplex (PDB: 7CM3) with the NALCN and FAM155A subunits in the 4-component NALCN channelosome (PDB: 7WJI). The overall structure is near identical except for the newly resolved II-III linker (red) of NALCN in this study. **(b)** The interface between the II-III linker of NALCN and the UNC79-UNC80 assembly. The interacting segments (named L1, H1, and H2) of NALCN and the HA repeats number of UNCs are labelled. **(c)** Sequence alignment of the II-III linker segments of NALCN among different species. The Uniprot IDs used for the alignments are: human NALCN: Q8IZF0; mouse NALCN: Q8BXR5; bovine NALCN: E1BK43; Narrow abdomen (*Drosophila* NALCN): A8JUW5; and NCA-2 (*C. elegans* NALCN): G5EDM1. **(d)** Sequence alignment of the II-III linker interacting regions of UNC80. The Uniprot IDs

used for the alignment are: human UNC80: Q8N2C7; mouse UNC80: Q8BLN6; bovine UNC80: F1MJ95; *Drosophila* UNC80: Q9VB11; and *C. elegans* UNC80: Q9XV66. **(e)** Sequence alignment of the II-III linker interacting region of UNC79. The Uniprot IDs used for the alignment are: human UNC79: Q9P2D8; mouse UNC79: Q0KK59; bovine UNC79: G3N0G4; *Drosophila* UNC79: Q06AJ1; and *C. elegans* UNC79: G5ECI9. Secondary structures are labelled above the sequence in **b-d**. The residues that may facilitate the interface interactions by structural analysis are indicated by triangles. Residues that are mutated and tested by electrophysiological experiments are indicated by red triangles.

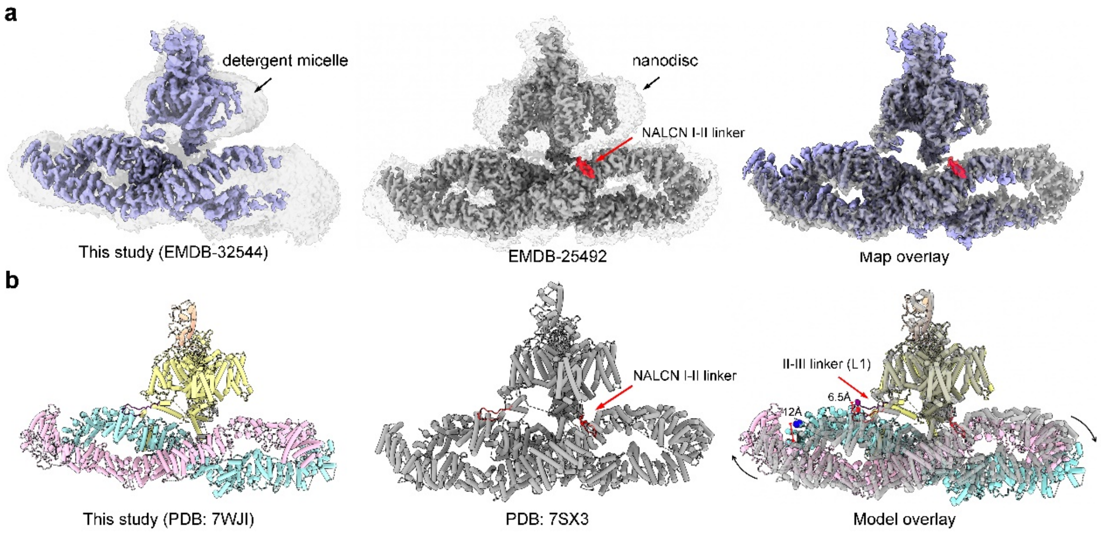

Supplementary Fig. S7. Comparison of the human NALCN channelosome structures. (a) Comparison of the overall EM maps of the NALCN channelosome in detergent micelle (this study, EMDB-32544) and in nanodisc (EMDB-25492). **(b)** Comparison of the overall models of the human NALCN channelosome. The I-II linker of NALCN revealed in PDB: 7SX3 is highlighted in red. Models are aligned against NALCN. A slight vertical rotation of the UNC79/UNC80 assembly between the two structures was observed and indicated by arrows. The shift distances of two selected sites NALCN-S647 and UNC80-Q2734 were measured.

Supplementary Table S1. Statistics for data collection and structure refinements.

	Human NALCN Channelosome (EMDB-32544) (PDB: 7WJI)	
**Data collection and processing**		
Microscope	FEI Titan Krios	
Magnification	81,000	
Voltage (kV)	300	
Detector	Gatan K3	
Electron exposure (e–/Å^2)	50	
Defocus range (μm)	-1.5 to -2.5	
Pixel size (Å)	1.087	
Symmetry imposed	C1	
Initial particle images (no.)	5,998,219	
**Refinement**	Overall	UNC80C-UNC79N masked region
Final particle images (no.)	174,294	383,924
Map resolution (Å)	4.5	3.3
FSC threshold	0.143	0.143
Map resolution range (Å)	4.1-15	2.9-15
Initial model used (PDB code)	7CM3	AlphaFold2 predicted structure
Model resolution (Å)	/	3.7
FSC threshold	/	0.5
Map sharpening *B* factor (Å^2)	157.8	87.2
Model composition		
Non-hydrogen atoms	41,175	15,879
Protein residues	5,128	1,976
Ligand	0	0
B factors (Å^2)		
Protein	219.70	98.55
Ligand	/	/
R.m.s. deviations		
Bond lengths (Å)	0.007	0.006
Bond angles (°)	1.461	1.299
Validation		
MolProbity score	1.82	1.56
Clashscore	7.55	4.90
Poor rotamers (%)	0.28	0.06
Ramachandran plot		
Favored (%)	93.87	95.63
Allowed (%)	6.11	4.37
Disallowed (%)	0.02	0

Supplementary Table S2. Summary of the NALCN channelosome modelling.

Subunit	Length (aa) /Uniprot ID	Modelled Regions /Coverage	Modeling method	Resolution (Å)
**NALCN**	1738/ Q8IZF0	Repeat I (31-322)<br>I-II linker (323-336; 373-382)<br>Repeat II (383-599)<br>II-III linker (600-617; 626-669; 714-737; 801-836; 846-880)<br>Repeat III (881-1156)<br>III-IV linker (1157-1209)<br>Repeat IV (1210-1447)<br>CTD (1448-1584)<br>80.2%	PDB: 7CM3<br><br>*De novo* building for III-IV linker segments	4.5-12
**FAM155A**	458/ B1AL88	192-382<br>41.7%	PDB: 7CM3	4.5-12
**UNC80**	3258/ Q8N2C7	HA repeats:<br>HA1 (20-73), HA2 (74-130), HA3 (166-230), HA4 (320-364), HA5 (653-698), HA6 (785-835), HA7 (836-865), HA8 (866-940), HA9 (941-960; 1177-1193), HA10 (1194-1234), HA11 (1235-1359), HA12 (1446-1503,1555-1568), HA13 (1569-1621), HA14 (1622-1669), HA15 (1845-1883), HA16 (1884-1918), HA17 (1918-1961), HA18 (1962-1994; 2079-2115), HA19 (2116-2176), HA20 (2177-2221), HA21 (2222-2265), HA22 (2266-2308), HA23 (2309-2390), HA24 (2391-2423; 2478-2519), HA25 (2520-2577), HA26 (2578-2629), HA27 (2630-2648; 2666--2705), HA28 (2704-2750; 2882-2913), HA29 (2733-2751; 2787-2807), HA30 (2808-2845), HA31 (2846-2881)<br>FERM-like domain (1995-2078)<br>54.1%	AlphaFold2 predicted structure+ manually adjustment	2.9-15
**UNC79**	2635/ Q9P2D8	HA repeats:<br>HA1 (1-52), HA2 (53-170), HA3 (171-208), HA4 (209-253), HA5 (254-280), HA6 (439-519), HA7 (520-568), HA8 (569-617), HA9 (618-652), HA10 (653-700), HA11 (701-730; 794-814), HA12 (815-844; 860-880), HA13 (943-995), HA14 (996-1039), HA15 (1040-1073), HA16 (1074-1119), HA17 (1120-1154), HA18 (1155-1180; 1222-1232; 1268-1301), HA19 (1302-1317; 1332-1351), HA20 (1352-1370; 1391-1416), HA21 (1417-1434; 2020-2036), HA22 (2037-2090), HA23 (2091-2117), HA24 (2118-2173), HA25 (2174-2206), HA26 (2207-2251), HA27 (2252-2289), HA28 (2290-2377), HA29 (2378-2425; 2598-2635), HA30 (2426-2499), HA31 (2500-2548), HA32 (2549-2597)<br>N-terminal small domain (281-404)<br>55.5%	AlphaFold2 predicted structure+ manually adjustment	2.9-15
**CaM**	149/ P0DP24	N-lobe (5-21, 28-56, 62-76)<br>C-lobe (86-149)<br>83.9%	PDB: 6MUD	~10

Supplementary Video S1. Conformational dynamics of the UNC79-UNC80 assembly.

References

1 Punjani, A., Rubinstein, J. L., Fleet, D. J. & Brubaker, M. A. cryoSPARC: algorithms for rapid unsupervised cryo-EM structure determination. *Nat. Methods* **14**, 290-296 (2017).

2 Pettersen, E. F. *et al*. UCSF chimera - A visualization system for exploratory research and analysis. *J. Comput. Chem.* **25**, 1605-1612 (2004).

3 Pettersen, E. F. *et al*. UCSF ChimeraX: Structure visualization for researchers, educators, and developers. *Protein Sci.* **30**, 70-82 (2021).

4 DeLano, W. L. The PyMOL Molecular Graphics System on World Wide Web https://www.pymol.org. (2002).
